# Taking a close look at a large-pore channel

**DOI:** 10.7554/eLife.56114

**Published:** 2020-03-31

**Authors:** Pablo S Gaete, Jorge E Contreras

**Affiliations:** Department of Pharmacology, Physiology and Neuroscience, New Jersey Medical School, Rutgers, The State University of New JerseyNewarkUnited States

**Keywords:** pannexin, heptemeric channel, atp release, extracellular loop, ion selectivity, carbenoxolone, Human, *Xenopus*

## Abstract

The structure of pannexin 1, a channel protein with a large pore, has been determined for the first time.

**Related research article** Michalski K, Syrjanen JL, Henze E, Kumpf J, Furukawa H, Kawate T. 2020. The cryo-EM structure of pannexin 1 reveals unique motifs for ion selection and inhibition. *eLife* **9**:e54670. doi: 10.7554/eLife.54670

A cell relies on proteins called ion channels and transporters to allow various ions and molecules to enter and leave the cell. These proteins are embedded in the plasma membrane of the cell, and some of them form dynamic pores that can close and open, allowing both ions and molecules to pass through. To date, researchers have largely focused on ion channels with relatively selective and narrow pores through which only specific types of atomic ions can pass. However, channel proteins with pores that are large enough for molecules to pass through are attracting more attention. These large-pore channels participate in a variety of physiological functions because they allow molecules with important signaling functions, such as ATP and glutamate, to pass through them.

Pannexin 1 is a large-pore channel that has important roles in inflammation, pain, infertility, cancer progression and epilepsy. It shows selectivity for anions, but it may also allow the passage of molecules as large as ~1 kilodalton in molecular weight. However, a lack of structural information has limited our understanding of how this and other large-pore channels work at the molecular level. Now, in eLife, Toshimitsu Kawate (Cornell University), Hiro Furukawa (Cold Spring Harbor Laboratory; CSHL) and colleagues – including Kevin Michalski (Cornell) and Johanna Syrjanen (CSHL) as joint first authors – report the first high-resolution structure of the pannexin 1 channel, obtained using cryo-electron microscopy (Michalski et al., 2020).

Michalski et al. show that the pannexin 1 channel has a unique architecture amongst eukaryotic channels, with seven subunits arranged around a large central pore (Figure 1). This contradicts previous studies that suggested that the pannexin 1 channel would be hexameric. The pore has three constriction sites, with the one in the extracellular region of the protein being the narrowest. This detail makes it likely that this constriction site acts as the main size-exclusion barrier, since its width could stop larger molecules from entering the pore. In this narrow extracellular region, the side chains of the tryptophan at position 74 of each subunit interact with the arginine at position 75 of the adjacent subunit, lining the pore. Arginine’s positive charge could repulse other positively charged molecules, potentially giving the channel its anion selectivity.

**Figure 1.**
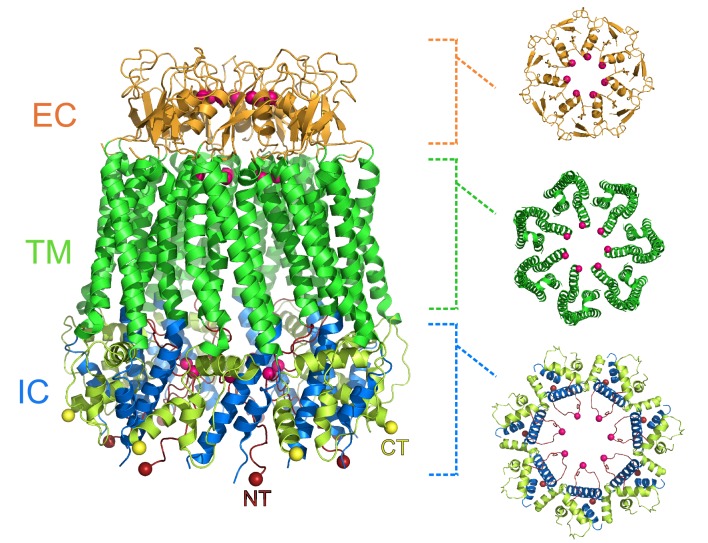
Heptameric structure of the pannexin 1 channel. Side view (left) of the pannexin 1 structure resolved by Michalski et al., and top views of the extracellular region (EC; top right), the transmembrane region (TM; middle right), and the intracellular region (IC; bottom right). The arrangement of seven subunits to form the channel is clearly visible in the structure. Each of the three regions shown in the top views contains a constriction site in the pore that runs through the center of the protein, and the amino acid residues involved in the constriction sites are represented as pink spheres. Protein data bank ID: 6VD7. CT: C-terminus (yellow); NT: N-terminus (red).

Mutating these arginine and tryptophan residues in all the subunits of the channel shows that their interaction, and particularly the presence of the arginine, are required for anion selectivity. These results are consistent with previous findings obtained by functional approaches (Ma et al., 2012). Both amino acids are highly conserved in different species, suggesting that selectivity for atomic anions could play an essential role in cell physiology, in addition to molecular transport.

Despite pannexin 1 being different in its amino acid sequence to other large-pore channels, including innexins and connexins, their topologies are quite similar: all have four transmembrane segments, two extracellular loops and one intracellular loop. Additionally, both their N-terminal and C-terminal regions are inside the cell. Consistent with this, the transmembrane segments of pannexin 1 almost overlap with the transmembrane segments of other large-pores channels. However, the structure of pannexin 1 shows substantial differences in the spatial conformation of the extracellular loops. This conformation may underlie specificity for two mechanisms that determine a channel’s activity. The first is gating, or how a channel changes its conformation to open and close the pore to allow atomic ions and other molecules through. The second is permeation, which determines how easily these molecules flow through the open pore.

Michalski et al. used their structural data to investigate the mechanisms through which carbenoxolone, one of the most widely used pannexin 1 inhibitors, blocks the channel. The amino acid residues involved in carbenoxolone sensitivity (identified in Michalski and Kawate, 2016) were located in a groove where the two extracellular loops interact, near the narrowest part of the pore. These structural insights could lead to the rational development of new, more specific, and potentially therapeutic drugs.

As with previous structural studies of large-pore channels, Michalski et al. could not resolve the full N- and C-terminal domains of pannexin 1, which suggests that these regions are flexible (Oshima et al., 2016; Kasuya et al., 2018; Michalski et al., 2020; Syrjanen et al., 2020). The importance of the N- and C-terminals has been shown through functional studies by either mutating the N-terminus, which alters gating and permeability (Michalski et al., 2018); or truncating the C-terminus using caspases, leading to a constitutively open channel (Chekeni et al., 2010; Sandilos et al., 2012).

This new evidence supports the need to obtain the full-length channel structures in open and closed conformations to understand both gating and permeability in large-pore channels. For example, the N-terminus is likely to further narrow the pore, making it difficult to envision how molecules such as ATP permeate pannexin 1 and other large-pore channels based on the partially solved structures reported to date. These issues are probably the next major challenges for structural biologists working on these channels.

Nonetheless, the structure resolved by Michalski et al. provides new insights into the biophysical properties of pannexin 1 channels. It can also serve as the basis for studies using complementary methodologies, including simulations of molecular dynamics and electrophysiology. In this view, the future of pannexin 1 research is auspicious, and several questions about the role of pannexin 1 in health and disease may be answered by integrating structural biology, biophysics and physiology.
